# Socket Preservation and Guided Bone Regeneration: Prerequisites for Successful Implant Dentistry

**DOI:** 10.7759/cureus.48785

**Published:** 2023-11-14

**Authors:** Yanko G Yankov

**Affiliations:** 1 Clinic of Maxillofacial Surgery, University Hospital "St. Marina", Varna, BGR; 2 Department of General and Operative Surgery, Medical University "Prof. Dr. Paraskev Stoyanov", Varna, BGR

**Keywords:** maxillofacial surgery, oral surgery, implantology, dental implant, bone augmentation, guided bone regeneration, socket preservation

## Abstract

Implant success is measured not only by implant survival but also by the long-term aesthetic and functional results. Implant placement should be prosthetically driven, with proper three-dimensional positioning for optimal support and stability of the tissues. Several procedures could be performed to ensure this requirement. While socket preservation (SP) is performed at the stage of tooth extraction, guided bone regeneration (GBR) takes place before or simultaneous to implant placement. The current review aims to summarize and discuss the procedures used for the preparation of the implant site, the preservation of the existing tissues, and their augmentation in cases of deficiency. An electronic search using Google Scholar, PubMed, and Scopus was conducted up to October 2023, in accordance with Preferred Reporting Items for Systematic Reviews and Meta-Analyses (PRISMA) guidelines. The review summarizes the current knowledge on SP and GBR as prerequisites for future implant placement. Their indications, advantages, and limitations have been thoroughly evaluated and some recommendations for further research have been suggested. Implant placement in sites with severe bone resorption is extremely challenging. It necessitates the application of different surgical techniques, especially augmentation procedures, including guided bone regeneration. The need for such procedures could be avoided or at least minimized by the execution of SP after tooth extraction or immediate/early implant placement.

## Introduction and background

The success of implant therapy is no longer measured only by implant survival but by the long-term aesthetic and functional results. Nowadays, implant placement should be prosthetically driven, with appropriate three-dimensional positioning for optimal support and stability of the surrounding hard and soft tissues [1].

The term “alveolar ridge preservation” refers to any procedure at the stage of tooth extraction aimed at minimizing the external resorption of the alveolar ridge and increasing bone formation in the dental socket [2]. The term was suggested in 1982 as “bone maintenance” [3]. Its synonyms are: “socket preservation” (SP), “ridge preservation”, and “socket grafting” [4].

According to a 2011 consensus [5], SP aims to support the residual hard and soft tissues, maintain a sufficient bone volume for optimal functional and aesthetic results, and facilitate the subsequent treatment procedures.

The procedure could be performed using different bone grafting materials, barrier membranes, or a combination of these. Recently, autologous platelet concentrates (APCs) have gained great popularity in guided bone regeneration (GBR) and SP [6-9]. APCs release a plethora of growth factors that promote hard and soft tissue healing. Procedures aimed at stimulating soft tissue preservation and even augmentation during tooth extraction have also been proposed. These include socket sealing methods. They utilize different barrier membranes [10-12] or soft tissue grafts [13-15] that cover the socket orifice and thereby protect the blood clot from disruption and the socket from infection. Similar techniques could be utilized even in cases of oroantral communications, which could benefit from both closure of the defect and preservation of the surrounding soft tissues [16,17].

Three categories of SP have been proposed regarding the resorption rate of the material used. The first category represents a long-term method for SP in cases where only prosthetic restoration without implant placement is planned. For this purpose, non-resorbable materials are used. The second category is SP with a medium duration. The materials used are slowly resorbable and preserve the bone volume for a long period. Implant placement could be performed after the stage of primary healing even in the presence of residual graft particles. This type of SP is indicated in cases when the implantation will be delayed significantly. The third category is a short-term SP that preserves the tissue volume during the initial healing phases. Implant placement is performed soon after that [18].

While SP and socket augmentation are performed at the stage of tooth extraction, guided tissue/bone regeneration and augmentation refer to procedures performed before or during implant placement.

GBR is a surgical procedure that requires a barrier membrane to guide bone deposition and the growth of soft tissues at the surgical site.

The basic principles of guided bone regeneration utilize barrier membranes in combination with bone grafting materials/ bone substitutes. They provide dimensional stability of the alveolar ridge [19,20]. The most commonly used biomaterials are allografts, xenografts, alloplastic materials, and autogenous bone.

There is a great variety of bone substitutes and researchers in the field are constantly trying to optimize their mechanical and biological properties [21]. This requires histological and histomorphometric testing of the newly developed materials to establish their safety and evaluate their tissue behavior [22]. Cone-beam computer tomography (CBCT) could be used as a reliable tool for the three-dimensional evaluation of the alveolar ridge [23,24].

GBR represents the concept of compartmentalization, suggested by Melcher [25]. It aims to prevent the migration of rapidly growing cells through the application of barrier membranes. They provide stability to the graft and prevent soft tissue collapse and undesired cell migration. Regarding the composition, barrier membranes could be made of synthetic materials such as polytetrafluoroethylene (PTFE), polyglycolic acid, polylactic acid, and trimethylene carbonate. Nowadays, resorbable membranes from natural origin are most commonly applied. Such a material is collagen. Some authors still consider the titanium-reinforced expanded PTFE membrane the gold standard in GBR [26].

This review aims to summarize, compare, and evaluate SP and GBR as methods for the preparation of the implant site, the preservation of the existing tissues, and their augmentation in cases of deficiency.

## Review

Materials and methods

An electronic search using Google Scholar, PubMed, and Scopus was conducted up to October 2023. The current review was conducted in accordance with the Preferred Reporting Items for Systematic Reviews and Meta-Analyses (PRISMA) guidelines.

The search included only articles in English, published from January 2018 to October 2023, and containing the following keywords: “guided bone regeneration”, “socket preservation”, and “dental implant placement”. The inclusion criteria were: articles that compare guided bone regeneration and socket preservation, and articles discussing their indications, advantages, and limitations. The exclusion criteria were: not full-text articles, abstracts, and citations; articles before 2018; articles in languages different from English.

This review was conducted in accordance with the Preferred Reporting Items for Systematic Reviews and Meta-Analyses (PRISMA) statement [27,28]. A comprehensive search in several databases (Google Scholar, PubMed, and Scopus) was conducted on October 10, 2023. The titles and abstracts were screened and evaluated for meeting the eligibility criteria. Duplicate records were removed and the rest of the articles were subjected to the inclusion and exclusion criteria.

Results

**Figure 1:**
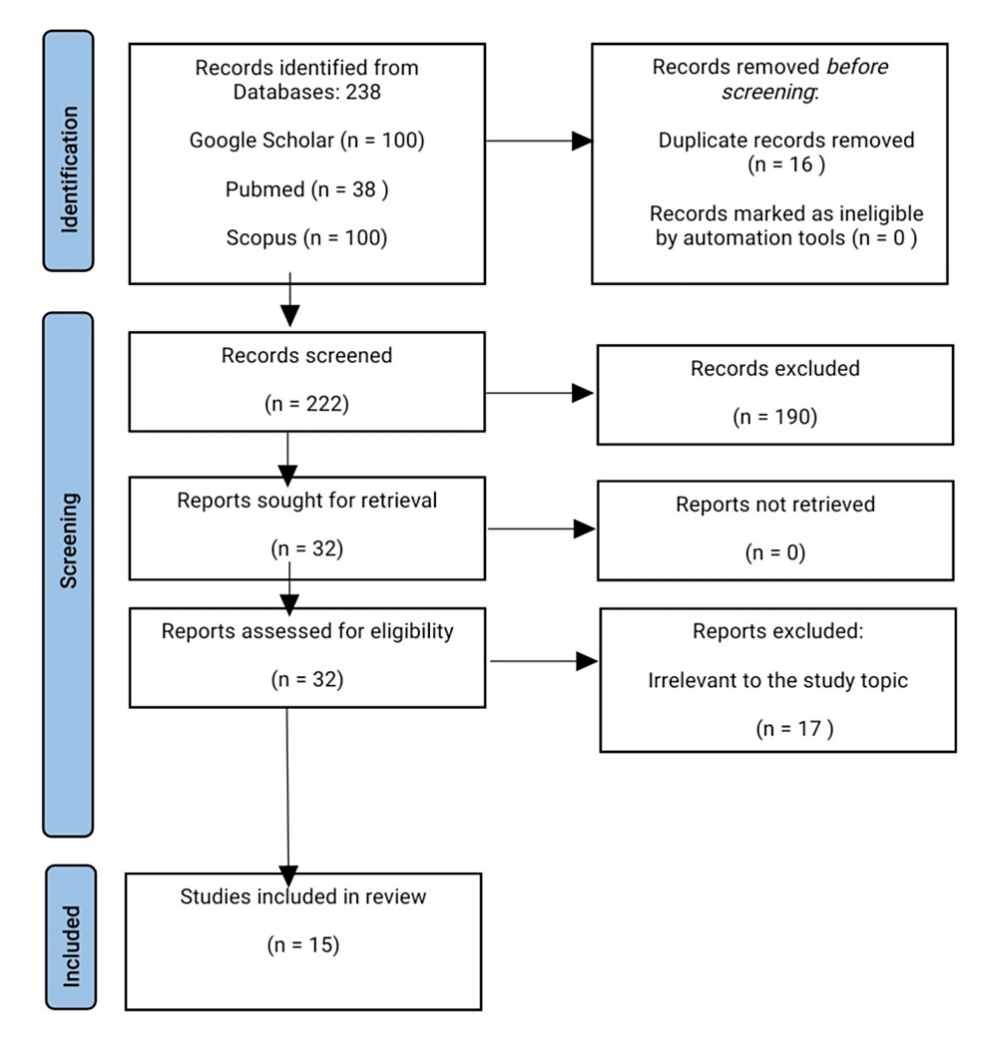
The PRISMA flow diagram PRISMA: Preferred Reporting Items for Systematic Reviews and Meta-Analyses

The initial search identified 14,144 potentially relevant studies (Google Scholar - 13,200; PubMed - 38; Scopus - 906). Then, the first 100 suggestions from both Google Scholar and Scopus and all 38 studies from PubMed were included for further evaluation. Sixteen duplicate records were excluded, and 222 records were screened and evaluated for eligibility. Finally, 15 relevant articles were included in the current study. The PRISMA flow diagram of the selection process is illustrated in Figure 1.

The characteristics of the review articles that met the eligibility criteria and were included in the current study are presented in Table 1.

**Table 1:** The characteristics of the review articles that met the eligibility criteria and were included in the current study GBR: guided bone regeneration; SP: socket preservation

Reference	Title	Year	Objective	Conclusions
Kalsi et al. [4]	Alveolar ridge preservation: why, when and how	2019	To discuss the current knowledge of SP methods and give recommendations for decision-making. The article evaluates the indications and limitations of treatment modalities in SP	SP successfully preserves bone volume and is beneficial for delayed implant placement. The method could eliminate the need for subsequent augmentation, such as sinus lift. Complete preservation of the ridge is unlikely to be achieved
Urban et al. [26]	Guided Bone Regeneration in Alveolar Bone Reconstruction	2019	Not clearly stated	GBR is a reliable and successful method for the reconstruction of atrophic alveolar ridges. However, the method is technically challenging and demanding
Jung et al. [29]	Alveolar ridge preservation in the esthetic zone	2018	To provide a clinical decision tree for SP in the esthetic zone	SP is not indicated (except from soft tissue defects) when implant placement is performed within 2 months after tooth extraction
Tonetti et al. [30]	Management of the extraction socket and timing of implant placement: Consensus report and clinical recommendations of group 3 of the XV European Workshop in Periodontology	2019	To provide evidence-based consensus statements and clinical guidelines	A substantial amount of data is available to assist the decision-making process. Further high-quality research is necessary for the development of clinical recommendations
Wessels et al. [31]	A 5-year cohort study on early implant placement with guided bone regeneration or alveolar ridge preservation with connective tissue graft	2020	To evaluate the 5-year outcome after early implant placement with GBR versus SP with late implant placement and connective tissue graft	Both methods demonstrated satisfactory long-term results. The aesthetic results were favorable in the SP-late implantation group but this should be interpreted with caution due to the selection bias and the additional connective tissue grafting
Al-Aroomi et al. [32]	Immediate implant placement with simultaneous bone augmentation versus delayed implant placement following alveolar ridge preservation: A clinical and radiographic study	2023	To evaluate the clinical and radiographic outcomes of immediate implant placement with GBR as compared to delayed implant placement after SP and to identify the potential risk factors	Immediate implants with GBR demonstrate better results than SP with delayed implant placement. However, the clinical outcomes and implant stability were similar in both groups
Mardas et al. [33]	Is alveolar ridge preservation an overtreatment?	2023	To discuss the objectives of SP and to determine where it can lead to favorable outcomes compared to unassisted socket healing	SP is an evidence-based method for extraction site management that can reduce the need for additional bone grafting and facilitate implant placement
Couso-Queiruga et al. [34]	Alveolar ridge preservation reduces the need for ancillary bone augmentation in the context of implant therapy	2022	To evaluate the efficacy of SP after tooth extraction compared with unassisted socket healing in reducing the need for additional bone augmentation	SP reduces the need for ancillary bone grafting at the time of implantation compared to unassisted healing
Lim et al. [35]	Ridge preservation in molar extraction sites with an open‐healing approach: A randomized controlled clinical trial.	2019	To determine the effect of SP in molar sites without primary flap closure	SP without primary flap closure in molar areas was effective in preserving the alveolar ridge and facilitated implant placement
Thoma et al. [36]	Explorative randomized controlled study comparing soft tissue thickness, contour changes, and soft tissue handling of two ridge preservation techniques and spontaneous healing two months after tooth extraction	2020	To compare two SP methods and unassisted socket healing in terms of soft tissue thickness, contour alterations, and soft tissue management two months following tooth extraction	SP provided more favorable soft tissue conditions compared to spontaneously healed sockets
Jonker et al. [37]	Soft tissue contour and radiographic evaluation of ridge preservation in early implant placement: A randomized controlled clinical trial	2021	To compare two SP methods and unassisted socket healing in terms of hard and soft tissue alterations	SP resulted in less bone resorption and reduced need for GBR procedures at early implant placement compared to unassisted socket healing
Barootchi et al. [38]	Ridge preservation techniques to avoid invasive bone reconstruction: A systematic review and meta-analysis: Naples Consensus Report Working Group C	2019	To evaluate and compare bone dimensional changes after SP and unassisted socket healing, and analyze the factors that have an impact on bone resorption	SP minimizes but cannot fully prevent bone resorption. The methods are most effective in the preservation of the alveolar ridge width
Simoni et al. [39]	Guided Bone Regeneration Effects on Bone Quantity and Outcomes of Dental Implants in Patients With Insufficient Bone Support: A Single-Center Observational Study	2023	To evaluate the effects of GBR procedures on bone quantity and short-term stabilization and survival of dental implants in cases with insufficient amount of bone	The application of GBR demonstrated a sufficient reduction in vertical depths between the healing abutments and the marginal bone, as well as stabilization of dental implants in patients with insufficient bone support.
Kinaia et al. [40]	Effect of guided bone regeneration on immediately placed implants: Meta-analyses with at least 12 months follow up after functional loading	2018	To evaluate the effect of GBR around immediate implant placement	A minimal difference in crestal bone level around immediate implants with and without bone graft was registered (in favor of GBR). Crestal bone level is better when bone graft is used in combination with a membrane compared to bone graft alone.
Cucchi et al. [41]	Statements and recommendations for guided bone regeneration: consensus report of the guided bone regeneration symposium held in Bologna, October 15 to 16, 2016	2019	To provide evidence-based clinical guidelines for GBR	GBR is a predictable method for bone augmentation. It provides adequate bone volume for the following implant placement. The success of the procedure depends on numerous patient-related factors and the correct planning.

Socket preservation is a beneficial technique for maintaining hard and soft tissue volume. It can preserve about 2 mm of both vertical and horizontal ridge dimensions [4].

The method aims to eliminate or at least reduce post-extraction ridge alterations, promote tissue healing, and facilitate prosthetically driven implant placement without additional grafting procedures. Jung et al. have suggested spontaneous healing or soft-tissue preservation in cases when implant placement is planned within 2 months after the extraction. If the implantation will be performed after that time, the type of SP depends on the size of the bone defect - soft and hard tissue preservation or only hard tissue preservation [29].

Sockets with thin buccal walls (<1.0-1.5 mm) demonstrate greater bone resorption after tooth extraction. A minimum healing time of three to four months is recommended after an SP procedure. According to the Consensus report and clinical recommendations of the XV European Workshop in Periodontology, SP is indicated in the aesthetic area irrespective of the subsequent restoration (implant-supported or tooth-retained); in extraction sites where major bone resorption is expected and implant placement could be jeopardized (thin and/or damaged buccal plates, posterior sites where further resorption could lead to implant proximity to the maxillary sinus or mandibular canal); in cases when implant placement will be significantly delayed [30].

Early implant placement is usually combined with guided regeneration of the buccal plate. The latter aims to provide adequate buccal height and thickness. This method requires two-stage surgery and allows for provisional implant restoration at about five months after the extraction. A major disadvantage of the procedure is its invasiveness, necessitating vertical incisions and the release of the periosteum. On the other hand, SP is minimally invasive and does not require flap elevation. However, it reduces bone formation in the socket and postpones implant placement. The surgery is one-stage and allows for provisional restoration at about five months after the extraction. Since SP does not allow for sufficient buccal augmentation, soft tissue grafts could be necessary. The latter increases patient morbidity. Furthermore, additional bone grafting is required in about 10% of the cases [31].

Similarly, Al-Aroomi et al. compare the clinical and radiological outcomes after immediate implant placement combined with GBR and SP with delayed implant placement [32]. The survival rate in both groups was 100%. The first group demonstrated some favorable results. However, the clinical outcomes, implant stability, and changes in bone level, thickness, and density were similar in both groups.

Socket preservation is a reliable method for extraction site management. It could reduce the need for additional bone augmentation, improve soft tissue thickness and contour, and facilitate implant placement in a prosthetically driven position. In addition, it does not have a negative impact on implant survival and success rates [33-37]. However, the methods cannot fully preserve the initial ridge dimensions.

Barootchi et al. have stated the following conclusions: even after SP, the resorption process continues and is most evident in the horizontal dimension of the ridge, followed by its buccal height, and SP with bone substitutes could reduce significantly but not eliminate the physiological cascade of post-extraction bone remodeling [38].

On the other hand, the use of GBR procedures at the stage of implant placement could successfully stabilize the implant in patients with bone deficiency [39] and allow for its correct prosthetic position. The technique is predictable and offers reproducible results.

Guided bone regeneration is indicated in the following cases: vertical/horizontal bone defects, bone fenestrations/dehiscence, and peri-implant defects. The main limitations of the GBR methods are that they demand great technical experience and excellent postoperative care [26]. Some authors demonstrate that there is a minimal difference in the crestal bone level when immediate implant placement is performed with and without bone substitutes [40].

Clinicians should note that the success of GBR depends on precise patient and defect analysis, exact surgical technique and flap manipulation, membrane fixation, and primary closure. Furthermore, soft-tissue management should be regarded as a significant step for long-term success [41].

Post-extraction bone resorption may necessitate the need for additional regenerative procedures during or before implant treatment. Socket preservation aims to preserve the available tissue volume, avoid the need for guided bone regeneration, and facilitate prosthetically guided implantation [4,29,42]. The procedure is beneficial for cases of delayed implant placement. Although it demonstrates promising results, complete tissue preservation has not been reported.

Socket preservation is indicated in the following cases: bone wall thickness less than 1-2 mm, involvement or loss of one or more alveolar walls, areas where progressive bone resorption could lead to communication with the maxillary sinus or mandibular canal, multiple extractions, increased aesthetic requirements, such as a high smile line or thin gingival biotype, where the risk of recession is increased [43], ridge countering for conventional prosthetic treatment, cases where a long delay in implantation is expected, e.g. very young patients, pregnancy, etc.

The majority of SP methods demonstrate positive results when compared to unassisted socket healing. However, some methods are effective only in vertical ridge preservation while others preserve both vertical and horizontal ridge dimensions [44].

Immediate implant placement with customized healing abutments is regarded as a successful method for SP with reduced healing time and excellent aesthetic outcomes [45]. The method should be considered in sites that do not require preliminary augmentation. Otherwise, the immediate implants should be combined with GBR.

Guided bone regeneration has been regarded as a reliable and successful method for bone augmentation. It has been demonstrated that both SP with delayed implant placement and immediate implant placement with GBR demonstrate comparable results in terms of clinical results and implant stability.

The need for GBR is determined by the amount and quality of the residual bone. The method is indicated in cases of primary bone deficiency and need for augmentation. Implant placement after GBR has demonstrated survival rates between 79 and 100% (the majority of studies have indicated more than 90% for at least one year after functional loading) [46]. However, there are still some challenging situations that necessitate the adoption of new strategies and materials with improved properties [47,48]. In addition, GBR is a more invasive procedure than SP. It is technically challenging and depends on various patient-related and surgery-related factors.

Limitations

This study has some potential limitations. It was conducted by only one researcher, which could have led to some subjective interpretations. In addition, records screening and evaluation were restricted to the first 100 suggestions of each database due to some flaws and weaknesses in their advanced searching. Furthermore, there is an insufficient amount of studies that compare both methods. Their indications, advantages, and limitations, as well as the correlation between SP and the subsequent need for GBR, need further assessment.

There are numerous methods for SP described in the literature. However, there is some study heterogeneity regarding the indications, outcomes, and success rates of these techniques. This could be due to several reasons: different criteria for patient selection; different surgical techniques; analysis based on different clinical measurements and paraclinical indicators; using different methods for diagnostics and statistical analysis, etc. Therefore, comparison between the methods is difficult and requires comparative studies with uniform criteria, goals, tasks, and methodology. 

## Conclusions

Implant treatment in areas with bone deficiency is extremely challenging. It necessitates the application of different surgical techniques, especially augmentation procedures. The present systematic review demonstrates that both SP and GBR should be regarded as key prerequisites for successful implant placement. The majority of studies suggest that SP is indicated in cases of delayed implant placement. The method could eliminate or at least reduce the need for additional augmentation at the stage of implant placement compared to unassisted socket healing. However, absolute preservation has not been reported. On the other hand, GBR has shown successful bone augmentation in areas with primary bone deficiency. Both SP with delayed implant placement and GBR with immediate implant placement reportedly demonstrate similar treatment outcomes. However, there is a lack of literature comparing SP and GBR, and, thus, further research is necessary to identify the indications, advantages, limitations, and success of both methods. Based on the current knowledge, the following guidelines for SP could be suggested: the procedure is indicated at post-extraction sites with thin buccal plates (<2 mm); areas with increased aesthetic risk; highly destroyed walls of the post-extraction sockets; multiple extractions; risk of involvement of some anatomical structures (the maxillary sinus, mandibular canal, etc.), and delayed implantation. Irrespective of the selected SP technique, the first step should always be an atraumatic tooth extraction.

Guided bone regeneration could be performed simultaneously to implant placement in areas with inadequate bone volume or as a preparation stage. The method allows for vertical and horizontal augmentation of atrophic jaws and thus ensures successful implantation in the correct three-dimensional position. Guided bone regeneration is indicated in the following cases: vertical/horizontal bone defects, bone fenestrations/dehiscence, and peri-implant defects. The main limitations of the GBR methods are that they demand correct planning, great technical experience, and excellent postoperative care.
